# Research Progress on Novel Lead Compounds for Central Nervous System Diseases

**DOI:** 10.3390/ph19060922

**Published:** 2026-06-11

**Authors:** Yuhan Qiao, Junwei Chen, You Zhou, Wenchao Shi

**Affiliations:** 1 State Key Laboratory of Resource Insects, College of Sericulture, Textile and Biomass Sciences, Southwest University, Chongqing 400715, China; hsqcb015817@email.swu.edu.cn (Y.Q.); chenjunwei2567@outlook.com (J.C.); zhouy701005@swu.edu.cn (Y.Z.); 2 Westa College, Southwest University, Chongqing 400715, China

**Keywords:** central nervous system diseases, therapeutic strategies, novel lead compounds, challenges and emerging strategies

## Abstract

Central nervous system (CNS) diseases, including ischemic stroke, Alzheimer’s disease, and Parkinson’s disease, impose a heavy socioeconomic burden worldwide. Current therapeutic strategies for CNS diseases primarily include modulating ion channels, inhibiting excitotoxicity, and applying anti-inflammatory, antioxidant, and anti-apoptotic approaches. However, existing drugs have not yet met the growing clinical demands. This paper summarizes novel lead compounds recently reported for CNS diseases and discusses the current challenges and emerging strategies in CNS drug development, aiming to provide a reference and scientific basis for future drug discovery and research.

## 1. Introduction

**Figure 1:**
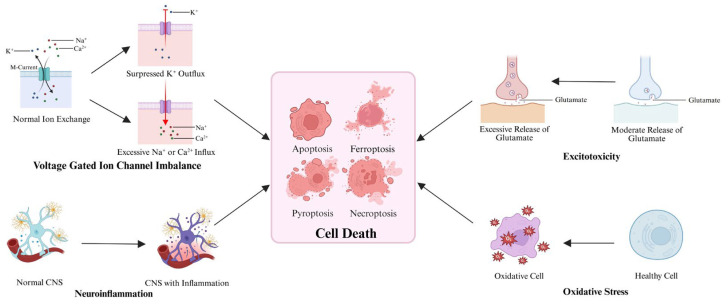
Core molecular mechanisms of CNS diseases. Created in Biorender. Yuhan Qiao (2026) https://BioRender.com/gpr0auo (accessed on 4 June 2026).

Central nervous system (CNS) diseases, including neurodegenerative diseases, ischemic stroke (IS), and traumatic brain injury (TBI), represent a major category of health issues primarily characterized by brain or spinal cord dysfunction. Their etiology is complex, involving multiple interactions among genetic, infectious, degenerative, and environmental factors [1,2,3]. The core molecular mechanisms underlying dysregulation of voltage-gated ion channels (VGICs), excitotoxicity, neuroinflammation, and oxidative stress ultimately lead to neuronal damage and various forms of cell death [4] (Figure 1). According to the World Health Organization, as of 2021, approximately one-third of the global population was affected by neurological diseases, and CNS diseases such as IS and Alzheimer’s disease (AD) ranked among the leading causes of disability adjusted life years [5]. Therefore, the development of drugs targeting CNS diseases is of critical importance. This review aims to systematically summarize recently reported novel lead compounds developed based on the core molecular mechanisms of CNS diseases, discuss the current challenges and emerging strategies in drug development, and provide a theoretical reference for the discovery of next-generation therapeutics. To achieve this goal, a systematic literature search was conducted in the Web of Science database, focusing on publications from the past three years. The lead compounds included in this review were selected based on their favorable pharmacological potential, including improvements in potency, selectivity, pharmacokinetic properties, or therapeutic efficacy. The most representative compounds are discussed in detail in the main text, whereas the remaining compounds are summarized in tables. Using the same database, this review further identifies and examines the major challenges in the field as well as the emerging and most representative strategies proposed to address them. Collectively, these findings provide an integrated overview of recent advances and future directions in CNS drug development.

## 2. Novel Lead Compounds Developed Based on the Core Molecular Mechanisms of CNS Diseases

### 2.1. VGIC Modulators

#### 2.1.1. VGICs and CNS Diseases

**Figure 2:**
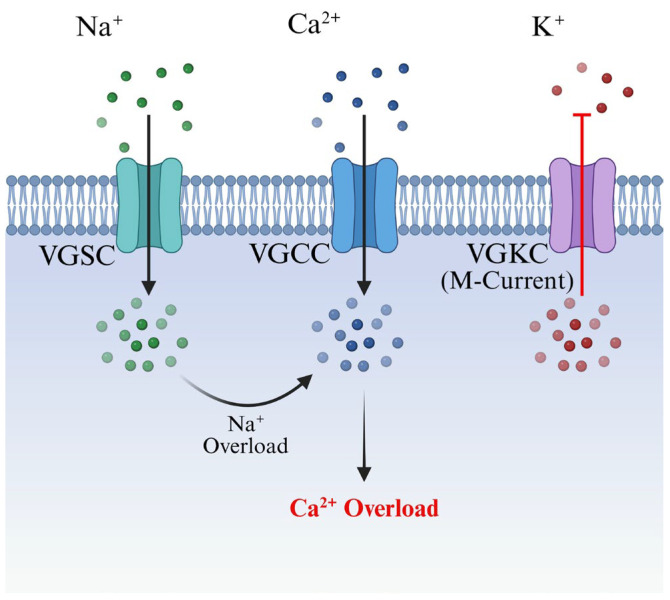
Molecular mechanism of VGIC imbalance. Created in Biorender. Yuhan Qiao (2026) https://BioRender.com/5p5b6ts (accessed on 4 June 2026).

VGICs are a class of integral membrane proteins that sense changes in transmembrane potential and mediate the transmembrane flux of water-soluble ions, thereby regulating processes such as neurotransmitter release, muscle contraction, and hormone secretion. They primarily include voltage-gated sodium channels (VGSCs), voltage-gated potassium channels (VGKCs), and voltage-gated calcium channels (VGCCs) [6]. Under pathological conditions in the CNS, sustained membrane depolarization can trigger abnormal sodium influx via VGSCs, leading to intracellular sodium overload and maintenance of a depolarized state. This subsequently activates VGCCs, leading to calcium overload and downstream injuries [7]. Furthermore, the M-current mediated by the Kv7 subfamily of VGKCs can strongly inhibit membrane depolarization and neuronal firing. In pathological states, excessive suppression of Kv7 channel activity leads to neuronal hyperexcitability, thereby contributing to the pathogenesis of CNS diseases such as epilepsy [8] (Figure 2).

#### 2.1.2. Novel VGIC Modulators

##### XPC-6591

Early sodium channel modulators often exhibit limited subtype selectivity and are frequently associated with CNS and cardiovascular side effects. Moreover, the high homology among sodium channel subtypes presents a significant challenge for the development of highly selective inhibitors. Johnson et al. [9] found that XPC-6591 was a highly selective Nav1.6 inhibitor that bound with high affinity to the voltage-sensing domain 4 (VSD4) of the human Nav1.6 channel, thereby modulating channel function (IC_50_ = 0.0056 μM). In the Scn8a^N1768D/+^ gain-of-function mouse model and the direct current multielectrode shock (DC-MES) model in wild-type (WT) mice, XPC-6591 exhibited potent anti-seizure activity at nanomolar concentrations, with EC_50_ values of 0.0047 μM and 0.01 μM, respectively. Notably, XPC-6591 demonstrated over 700-fold selectivity for Nav1.1 and 100-fold selectivity for Nav1.5. Compared with currently available clinical sodium channel inhibitors, XPC-6591 showed higher selectivity, thereby significantly reducing the risk of potential central nervous system and cardiac side effects. Furthermore, XPC-6591 was a brain-penetrant Nav1.6 inhibitor that exerted anti-epileptic effects at very low cerebral concentrations. As one of the most potent Nav1.6 inhibitors reported to date, XPC-6591 highlights the therapeutic potential of the VSD4-targeting strategy. Future studies are required to further elucidate its long-term safety and efficacy in additional disease models, thereby facilitating its clinical translation.

##### ICA-27243

The M-current is a critical outward potassium current mediated by Kv7.2/7.3 heterodimers, and its functional homeostasis is essential for suppressing abnormal neuronal discharge. ICA-27243 initially gained attention as a potential anti-epileptic drug; however, Padilla et al. [10] found that this compound also exerted broader neuroprotective effects by targeting potassium channels. Mechanistic studies revealed that ICA-27243 allosterically activated Kv7.2/7.3 channels by binding to the S1-S4 voltage-sensing domain of the Kv7.2 channel, thereby alleviating neuronal hyperexcitability. Masegosa et al. [11] further demonstrated that ICA-27243 (10 μM) increased neuronal survival by 21% in an organotypic spinal cord culture model of acute excitotoxicity. In vivo, ICA-27243 (10 mg/kg) also significantly attenuated the decline in neuromuscular function, preserved motor coordination, and decreased glial reactivity in SOD1^G93A^ transgenic mice. Moreover, following ICA-27243 treatment, the number of spinal motor neurons in SOD1^G93A^ mice increased approximately 1.75-fold, reaching 58% of the level observed in WT mice, and the onset of motor impairment was delayed by at least two weeks. This study not only provides the first evidence of the neuroprotective effect of ICA-27243 on spinal motor neurons but also highlights the importance of allosteric modulation of Kv7 channels as an intervention strategy to counteract neuronal hyperexcitability. However, although ICA-27243 also exhibited a trend toward reducing glial reactivity in SOD1^G93A^ transgenic mice during the experiment, this effect did not reach statistical significance. Therefore, its impact on glial cell activity warrants further investigation.

##### Magnolol

Recent studies have identified that cannabinoids possess antiepileptic properties. Magnolol, a cannabinoid-like compound derived from *Magnolia officinalis*, has attracted considerable attention due to its structural similarity to cannabinoids. Yip et al. [12] first reported that magnolol exerted anti-epileptic effects by inhibiting T-type calcium channel subtypes, with IC_50_ values of 4 μM, 5 μM, and 4 μM for Cav3.1, Cav3.2, and Cav3.3, respectively. This effect was achieved by directly inhibiting calcium currents rather than acting on cannabinoid CB1 or CB2 receptors. In Scn1a^+/−^ mice, magnolol (100 mg/kg) significantly increased the threshold for febrile seizures; in the maximal electroshock (MES) test, the same dose protected 90% of mice from hindlimb extension; and in Gabrb3^+/D120N^ mice, magnolol significantly reduced the frequency and duration of atypical absence seizures. Pharmacokinetic studies showed a brain-to-plasma concentration ratio of 3.55 for magnolol, indicating excellent brain permeability. This study demonstrates that magnolol is effective in both refractory epilepsy models described above. However, its efficacy against other seizure types, such as spontaneous generalized tonic–clonic seizures, has not yet been evaluated in these models. In addition, limitations including its low oral bioavailability remain to be addressed in future investigations.

Table 1 summarizes the effects of novel VGIC modulators, their molecular mechanisms, and their reported activities in various in vivo models for the treatment of CNS diseases.

**Figure 3:**
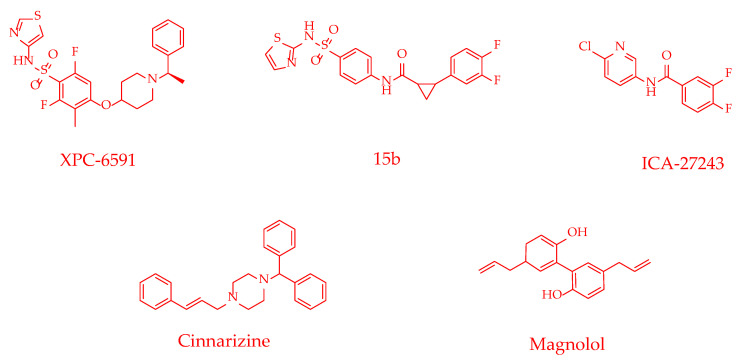
Chemical structures of novel VGIC modulators.

Figure 3 shows chemical structures of VGIC modulators listed in Table 1.

### 2.2. Excitotoxicity Inhibitors

#### 2.2.1. Excitotoxicity and CNS Diseases

**Figure 4:**
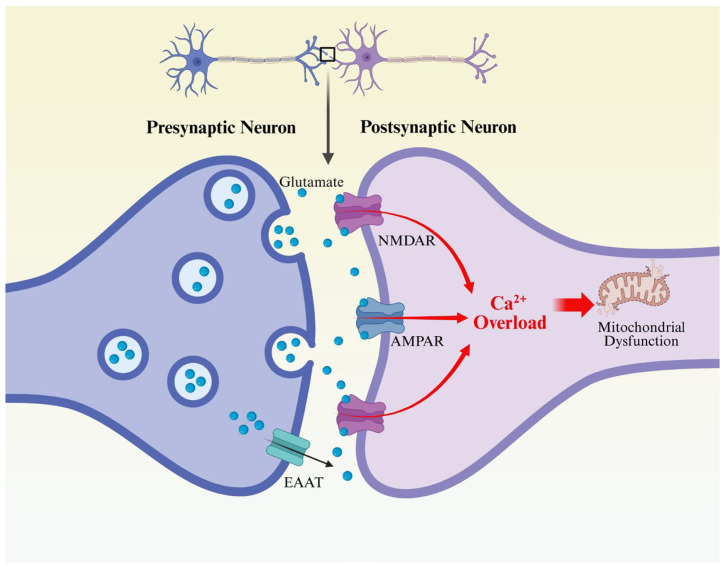
Molecular mechanism of excitotoxicity. Created in Biorender. Yuhan Qiao (2026) https://BioRender.com/zdvy1p5 (accessed on 5 June 2026).

Excitotoxicity refers to the pathological mechanism by which excessive or sustained stimulation of postsynaptic neuronal receptors by excitatory neurotransmitters leads to neuronal injury or death [15]. Glutamate is one of the principal excitatory neurotransmitters in the CNS, participating in over 90% of neuronal activation processes by binding to glutamate receptors on the postsynaptic membrane. Under pathological conditions, sustained neuronal depolarization leads to a massive release of glutamate from synaptic vesicles; simultaneously, energy depletion may reverse the transport direction of excitatory amino acid transporters (EAATs), resulting in extensive non-vesicular glutamate efflux [16,17]. Excessive glutamate stimulation induces intracellular calcium overload, activates downstream oxidative stress signaling pathways and the p38 mitogen-activated protein kinase (MAPK) signaling pathway, disrupts mitochondrial stability, and ultimately causes progressive and sustained neuronal death [18]. (Figure 4) Among the glutamate receptor family, the roles of the ionotropic glutamate receptors, specifically the N-methyl-D-aspartate receptor (NMDAR) and the α-amino-3-hydroxy-5-methyl-4-isoxazolepropionic acid receptor (AMPAR), are particularly crucial [19].

The NMDAR is a ligand-gated, voltage-dependent ion channel widely distributed throughout the CNS, primarily consisting of two GluN1 subunits and two GluN2 subunits; the latter can be further classified into four subtypes: GluN2A, GluN2B, GluN2C, and GluN2D [20]. Although NMDARs contribute to excitotoxicity, differences in subunit composition and cellular localization confer dual functional effects. It has been proposed that the activation of GluN2A-containing NMDARs may exert neuroprotective effects, whereas the activation of GluN2B-containing NMDARs tends to induce neuronal death; however, inconsistencies remain among relevant research findings [21]. The AMPAR is a tetrameric ion channel composed of four subunits (GluA1-GluA4), and different combinations of these subunits confer its structural diversity [22]. Under certain conditions, AMPARs can mediate neurotoxic calcium influx, and their structural variations or functional overactivation are closely associated with the pathological progression of various CNS diseases.

#### 2.2.2. Novel Excitotoxicity Inhibitors

##### 2,3,5,4′-Tetrahydroxystilbene-2-O-β-D-Glucoside

2,3,5,4′-Tetrahydroxystilbene-2-O-β-D-glucoside (THSG) is the primary active component of the traditional Chinese medicine *Polygonum multiflorum*, which has been used clinically as a tonic and anti-aging medicine for over a millennium. Liu et al. [21] demonstrated that THSG could form hydrogen bonds with residues such as Gly128 and Arg292 in the GluN2B subunit and engage in Pi-Alkyl interactions with Ala263 and Met132 residues, thereby stably occupying the docking pocket (binding energy = −5.2 kcal/mol). This binding subsequently activated the GluN2B–CaMKII–ERK1/2 signaling pathway, enhanced PINK1/PARK2-mediated mitophagy, inhibited neuronal apoptosis, and alleviated cerebral ischemia–reperfusion injury (CIRI) in transient middle cerebral artery occlusion/reperfusion (tMCAO/R) rats. Experiments confirmed that THSG (40 mg/kg) could significantly reduce cerebral infarct volume in tMCAO/R rats; compared with the positive control drug salvianic acid A sodium, rats in the THSG group exhibited lower neurological deficit scores and greater neurological functional improvements. This study reveals that THSG exerts neuroprotective effects through a GluN2B-dependent mechanism, providing a new strategy for developing NMDAR-targeted neuroprotectants based on natural products. However, the efficacy of this compound has thus far been validated only for a single type of neuronal mitophagy. Its effects on other mitophagy pathways and in other cell types remain to be investigated. Moreover, the pharmacokinetic characteristics and clinical translational potential of THSG warrant further evaluation.

##### L-Theanine

L-Theanine is a non-protein amino acid present in tea leaves that has attracted considerable attention for its potential to modulate nervous system functions. Park et al. [23] demonstrated that this compound exerted a central protective effect in TBI through multi-targeted mechanisms. As a structural analog of glutamate, L-theanine could bind to and inhibit AMPARs, specifically suppressing the overactivation of the GluA1 subunit, thereby directly antagonizing excitotoxicity. It exhibited therapeutic efficacy comparable to that of the AMPAR antagonist NBQX. Furthermore, L-theanine could enter astrocytes, promote glutathione synthesis, thereby enhancing endogenous antioxidant defense capacity. In a rat model of controlled cortical impact—traumatic brain injury (CCI-TBI), treatment with L-theanine (200 mg/kg) significantly attenuated zinc accumulation, neuronal death, and cognitive deficits in the hippocampus, accompanied by improvements in markers of oxidative stress and neuroinflammation. These results provide preclinical evidence for developing L-theanine as a neuroprotectant; its natural origin and favorable safety profile constitute distinct advantages. Nevertheless, future studies are required to elucidate its effects on neuron–astrocyte interactions in vivo and to explore additional underlying molecular mechanisms.

Table 2 summarizes the effects of novel excitotoxicity inhibitors, their molecular mechanisms, and their reported activities in various in vivo models for the treatment of CNS diseases.

**Figure 5:**
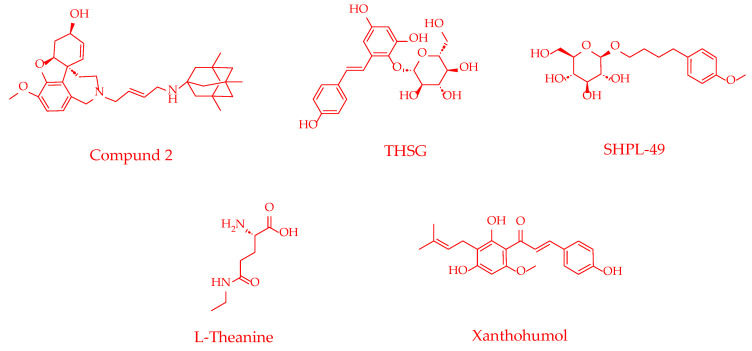
Chemical structures of excitotoxicity inhibitors.

Figure 5 shows chemical structures of excitotoxicity inhibitors listed in Table 2.

### 2.3. Anti-Inflammatory Agents

#### 2.3.1. Inflammation and CNS Diseases

**Figure 6:**
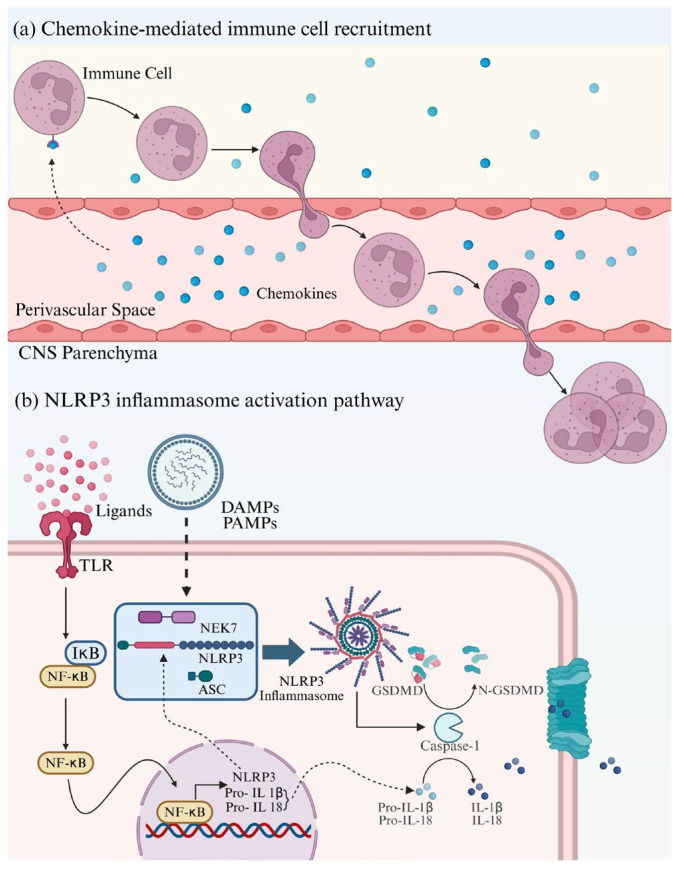
Molecular mechanisms of CNS neuroinflammation. (**a**) Chemokine-mediated immune cell recruitment. Solid arrows represent the process of immune cell infiltration; dashed arrows represent cytokines crossing the perivascular space to bind to immune cells. (**b**) NLRP3 inflammasome activation pathway. Solid arrows represent direct effects; dashed arrows represent indirect effects. Created in Biorender. Yuhan Qiao (2026) https://BioRender.com/976pf4d (accessed on 4 June 2026).

CNS Neuroinflammation refers to the process in which immune cells infiltrate the CNS and activate resident microglia and astrocytes [28]. A moderate inflammatory response is protective, contributing to pathogen clearance and tissue repair; however, when the inflammatory response becomes uncontrolled, chronic, and excessive, it drives various neurological diseases [29]. Under pathological conditions, NLRP3 inflammasomes are transcriptionally upregulated via the TLR/NF-κB signaling pathway and subsequently triggered by pathogen-associated molecular patterns (PAMPs) and damage-associated molecular patterns (DAMPs) to assemble and activate caspase-1. On the one hand, activated caspase-1 promotes the maturation of IL-1β and IL-18; on the other hand, it cleaves gasdermin D (GSDMD), allowing its N-terminal domain to translocate to the cell membrane and form pores, thereby mediating pyroptosis and releasing the pro-inflammatory cytokines IL-1β and IL-18 [30]. Simultaneously, during neuroinflammation, endothelial cells of the blood–brain barrier (BBB) upregulate adhesion molecules and transport chemokines, which recruit peripheral immune cells to migrate and infiltrate into the CNS, further exacerbating neuroinflammation [31] (Figure 6). Therefore, targeting the inhibition of NLRP3 inflammasome assembly and activation, blocking the release of proinflammatory cytokines, and modulating chemokines or their receptor signaling have emerged as key strategies for intervening in CNS neuroinflammation.

#### 2.3.2. Novel Anti-Inflammatory Agents

##### Talniflumate

In PD, upregulation of alanine-serine-cysteine transporter 2 (ASCT2) in astrocytes promotes its interaction with the NLRP3 inflammasome, thereby exacerbating neuroinflammation. Talniflumate was originally developed as an anti-inflammatory analgesic. Through virtual screening of an FDA-approved drug library, Liu et al. [32] identified that it could specifically target ASCT2. By specifically blocking the physical interaction between ASCT2 and NLRP3, this compound inhibited NLRP3 inflammasome assembly and activation, thereby alleviating neuroinflammation. In the MPTP-induced mouse model, talniflumate (50 mg/kg) efficiently crossed the BBB, attenuated neuroinflammation, protected dopaminergic neurons, and significantly improved the motor function. This study reveals a novel mechanism by which astrocytic ASCT2 participates in NLRP3 inflammasome activation, influencing the glial inflammatory response and PD progression. Moreover, target-based screening identified the ASCT2 inhibitor talniflumate, providing a new therapeutic strategy and a candidate drug for PD treatment. Nevertheless, further experimental validation is warranted to elucidate the precise binding sites between ASCT2 and NLRP3, as well as to assess the efficacy of Talniflumate in additional PD models.

##### Metformin

As a classic first-line oral hypoglycemic drug, metformin (MF) has been increasingly recognized for its novel pharmacological effects. In recent years, MF has shown neuroprotective potential in neurological diseases. Akter et al. [33] established a mouse stroke model with BBB disruption superimposed on tobacco smoke (TS) exposure to investigate the potential therapeutic effects of MF on TS-induced stroke-related neural damage. The results indicated that MF primarily activated the Nrf2-ARE antioxidant signaling pathway and inhibited the NF-κB inflammatory pathway, significantly reducing the release of pro-inflammatory factors such as TNF-α and IL-1β in primary cultured neurons and astrocytes in vitro. Further in vivo experiments confirmed that MF intervention (200 mg/kg) significantly attenuated the neuroinflammatory response in adolescent mice following stroke and TS exposure, downregulated NF-κB expression in brain tissue, and decreased the levels of inflammatory factors, including TNF-α, IL-1β, MCP-1, and MIP-2. This study is the first to systematically explore the direct anti-neuroinflammatory effects of MF on primary neurons and astrocytes under conditions of TS and IS. Furthermore, research on the impact of TS exposure on the Nrf2-ARE signaling pathway in adolescent mice has been rarely reported; this study fills the gap in this field, providing a new animal model basis for research on smoking-related neuroinflammation in adolescents. A limitation of this study is that neither the therapeutic time window for MF administration nor the optimal dosage required to maintain adequate cerebral concentrations has been established, warranting further experimental validation.

##### 3-Monothiopomalidomide

3-Monothiopomalidomide (3-MP) is a novel immunomodulatory imide drug derived from pomalidomide. Hsueh et al. [34] found that it bound to cereblon (CRBN), a key adaptor protein of the E3 ubiquitin ligase complex, thereby interfering with CRBN-mediated inflammatory signal transduction (IC_50_ = 0.21 μM), demonstrating significantly superior efficacy compared to pomalidomide (IC_50_ = 2.38 μM), while not triggering the downstream degradation of SALL4 and Aiolos proteins, thus avoiding associated adverse reactions. 3-MP (10–60 μM) effectively reduced pro-inflammatory markers in LPS-stimulated murine macrophages and microglia. In in vivo experiments, 3-MP (26.5 mg/kg and 53 mg/kg) significantly reduced pro-inflammatory cytokine and chemokine levels in both plasma and brain of LPS-treated mice, without affecting the anti-inflammatory cytokine IL-10. Pharmacokinetic studies revealed that 3-MP readily crosses the BBB following intraperitoneal injection, with a brain-to-plasma concentration ratio of 0.44–0.47 (2.65–26.5 mg/kg), indicating good brain penetrability. In the CCI-TBI mouse model, 3-MP (13.23 mg/kg and 26.47 mg/kg) markedly alleviated behavioral deficits and inhibited astrocyte and microglial activation. In summary, 3-MP is a drug candidate possessing both brain penetrability and anti-inflammatory activity. Compared with pomalidomide, it exhibits lower toxicity and holds promise for the treatment of neuroinflammation-related diseases. Future studies are needed to evaluate its long-term safety profile and further assess its teratogenic potential across different experimental models.

Table 3 summarizes the effects of novel anti-inflammatory agents, their molecular mechanisms, and their reported activities in various in vivo models for the treatment of CNS diseases.

**Figure 7:**
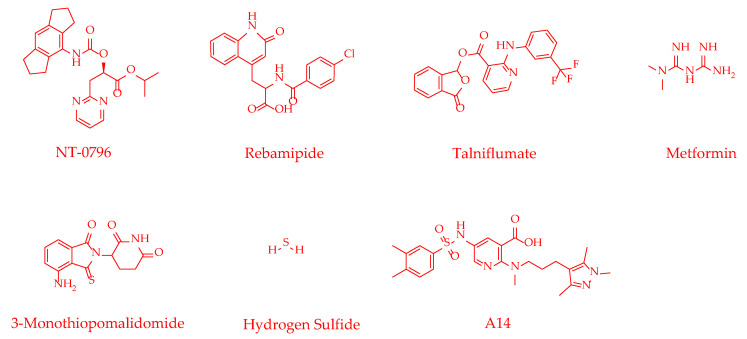
Chemical structures of anti-inflammatory agents.

Figure 7 shows chemical structures of anti-inflammatory agents listed in Table 3.

### 2.4. Antioxidants

#### 2.4.1. Oxidative Stress and CNS Diseases

**Figure 8:**
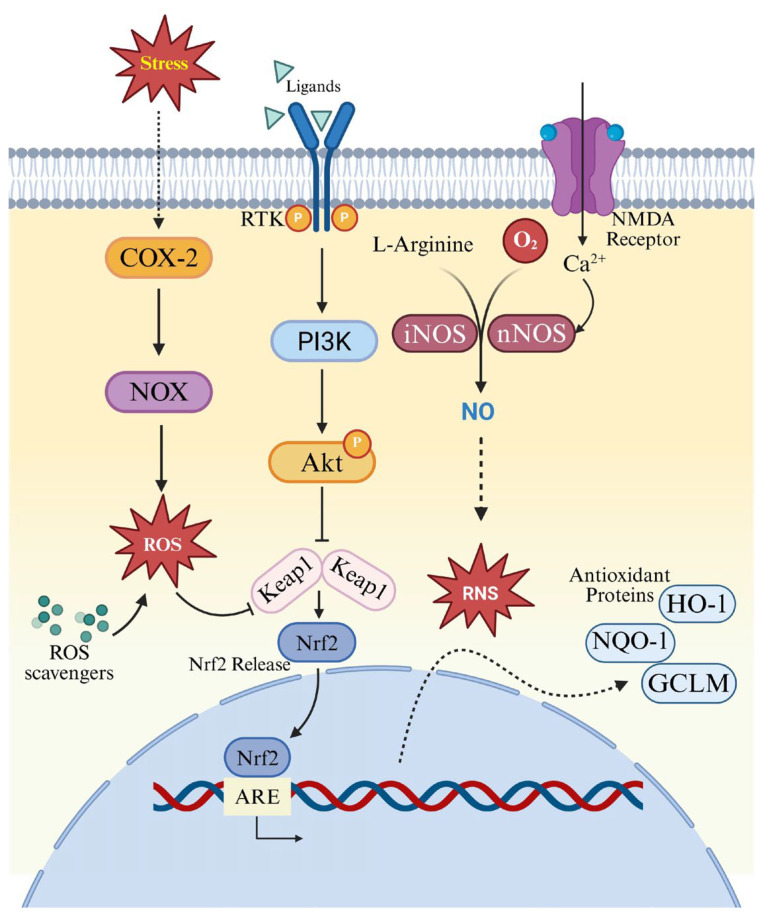
Molecular mechanisms of the intracellular antioxidant system. Arrows indicate stimulatory interactions, with solid arrows representing direct effects and dashed arrows representing indirect effects; bars denote inhibitory effects. Created in Biorender. Yuhan Qiao (2026) https://BioRender.com/zdw19kw (accessed on 4 June 2026).

The essence of oxidative stress lies in an imbalance between pro-oxidant and antioxidant homeostasis, where the rate of reactive oxygen species (ROS) and reactive nitrogen species (RNS) generation exceeds the scavenging capacity of the antioxidant defense system. The CNS has a remarkably high energy demand, and its ATP is primarily generated through mitochondrial oxidative phosphorylation. While this process is essential for maintaining normal function, it inevitably generates ROS. Furthermore, superoxide anions produced via the activation of NADPH oxidase (NOX) can also be converted into ROS [40]. Low to moderate concentrations of ROS play important physiological roles, but under oxidative stress conditions, excessive ROS is highly detrimental [41]. Under pathological conditions, nitric oxide (NO) produced by certain nitric oxide synthases (NOSs) can trigger an explosive increase in RNS, constituting a critical mechanism that aggravates oxidative damage in the CNS [42]. When the rate of ROS/RNS production surpasses the clearance capability of endogenous enzymatic systems and small-molecule antioxidants, the resulting excess free radicals attack proteins, lipids, and DNA, ultimately leading to neuronal damage and functional decline [43]. To counteract oxidative stress, cells phosphorylate and activate the transcription factor Nrf2 via the PI3K/Akt signaling pathway, promoting its dissociation from Keap1 and subsequent nuclear translocation. This initiates the transcription of antioxidant enzyme genes, such as heme oxygenase-1 (HO-1), thereby constituting the core endogenous defense system. Simultaneously, small-molecule antioxidants can interrupt free radical chain reactions to decrease ROS formation [44,45] (Figure 8). Based on these mechanisms, targeted activation of the PI3K/Akt/Nrf2 pathway, inhibition of NOX and NOS activities, and direct ROS scavenging have emerged as vital strategies for developing central neuroprotective agents.

#### 2.4.2. Novel Antioxidants

##### Gomisin N

Gomisin N (GN) is a natural lignan compound isolated from the traditional Chinese medicine *Schisandra chinensis*. It has attracted widespread attention due to its diverse pharmacological activities, including hepatoprotective, anti-inflammatory, and antioxidant effects. In recent years, its neuroprotective potential has also been investigated. Li et al. [44] found that in the mouse model of middle cerebral artery occlusion/reperfusion (MCAO/R), GN (30–90 mg/kg) could significantly attenuate neuronal damage, reduce cerebral infarct volume, and improve neurological deficits. Mechanistic analysis revealed that GN activated the PI3K/Akt/mTOR signaling pathway by promoting the phosphorylation of key proteins PI3K, AKT, and mTOR (binding energy of GN with these proteins: PI3K = −9.9 kcal/mol; Akt = −8.5 kcal/mol; mTOR = −8.0 kcal/mol), thereby inhibiting overactivated autophagy and reducing neuronal death. Molecular docking studies demonstrated a strong binding affinity between GN and PI3K, and the protective effect was reversed by the PI3K inhibitor LY294002, further confirming the critical role of this pathway. This study is the first to elucidate a novel neuroprotective mechanism of GN in cerebral ischemia by modulating autophagy, providing important preclinical evidence for its further development as a candidate anti-IS agent. Future research should further explore its multi-target synergistic mechanisms and in vivo pharmacokinetic properties.

##### Dl-3-n-Butylphthalide and Its Derivatives

Dl-3-n-butylphthalide (NBP) can exert neuroprotective effects in various CNS diseases by activating the Nrf2 signaling pathway to alleviate oxidative stress and neuroinflammation. Liu et al. demonstrated that in a nitroglycerin (NTG)-induced chronic migraine mouse model, NBP (30 mg/kg) significantly reduced oxidative stress and inflammation, and these therapeutic effects were markedly attenuated following administration of the Nrf2 inhibitor ML385 [46]. Compared with edaravone-dexborneol, NBP showed greater effects in reducing cerebral infarct volume, restoring cerebral blood flow, and scavenging oxidative stress factors [47]. Furthermore, Li et al. [48] synthesized a hybrid compound 8a by conjugating NBP with ligustrazine (TMP) via a triazole linkage. This hybrid, bound to Keap1, promoted Nrf2 dissociation and nuclear translocation and upregulated the production of downstream antioxidant factors, thereby reducing intracellular ROS levels and effectively protecting neuronal mitochondria. In the OGD/R-induced SH-SY5Y cell injury model, a low concentration of 8a (6.25 μM) achieved a protection rate of 75.6% without detectable cytotoxicity. It was demonstrated to activate the Nrf2 pathway and reduce markers of cell injury, oxidative stress, and apoptosis, showing greater efficacy than NBP. In the rat MCAO/R model, 8a (20 mg/kg) significantly reduced cerebral infarct volume, improved mNSSs, and enhanced cerebral blood flow, displaying superior therapeutic efficacy compared with NBP at the equivalent dose. In conclusion, NBP and its derivatives show promising potential for the treatment of various CNS diseases and warrant focused attention in future research, but further studies are required to characterize their pharmacokinetic profiles.

##### Crocetin

Crocetin is a natural carotenoid that can be extracted directly from saffron (*Crocus sativus*) and is also the intestinal metabolite of crocin, the principal water-soluble active constituent of saffron, exhibiting excellent BBB permeability. Wu et al. [49] found that this compound could antagonize parthanatos (PARP-1-dependent programmed cell death) during IS through a dual mechanism. In the early phase, crocetin inhibited the interaction between protein kinase Cζ (PKCζ) and the NOX subunit p47^phox^, thereby blocking the NOX2 complex assembly and activation, and markedly suppressing the ROS burst. In the late phase, crocetin directly bound to hexokinase-I (HK-I), specifically targeting key residues such as Leu31 and His373, which inhibited E3 ubiquitin ligase RNF146-mediated PARylation and ubiquitination degradation of HK-I, maintained the binding of HK-I to mitochondria, protected mitochondrial function, and prevented the nuclear translocation of apoptosis-inducing factor (AIF). The study demonstrated that crocetin pretreatment could elevate the neuronal survival to a level comparable to that achieved with the selective PARP inhibitor PJ34, indicating that crocetin effectively promoted cell survival through the anti-parthanatos pathway. In vivo experiments confirmed that crocetin (40 mg/kg) reduced the cerebral infarct volume in rats with pMCAO from 43.11% to 12.91%, and the mNSS decreased from 13.2 to 9.4. These findings not only reveal the potential of crocetin as a multi-target neuroprotective agent but also provide important insights for the development of novel anti-IS drugs targeting the NOX2-HK-PARP axis. However, the pharmacokinetic properties, clinically applicable dosage, and long-term safety of this compound still require further comprehensive investigation.

Table 4 summarizes the effects of novel antioxidants, their molecular mechanisms, and their reported activities in various in vivo models for the treatment of CNS diseases.

**Figure 9:**
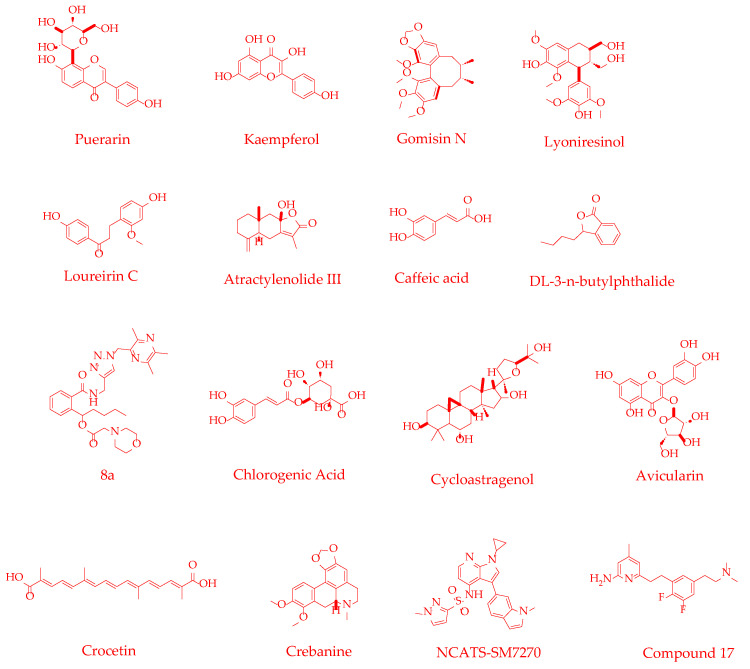
Chemical structures of antioxidants.

Figure 9 shows chemical structures of antioxidants listed in Table 4.

### 2.5. Cell Death Inhibitors

#### 2.5.1. Cell Death and CNS Diseases

**Figure 10:**
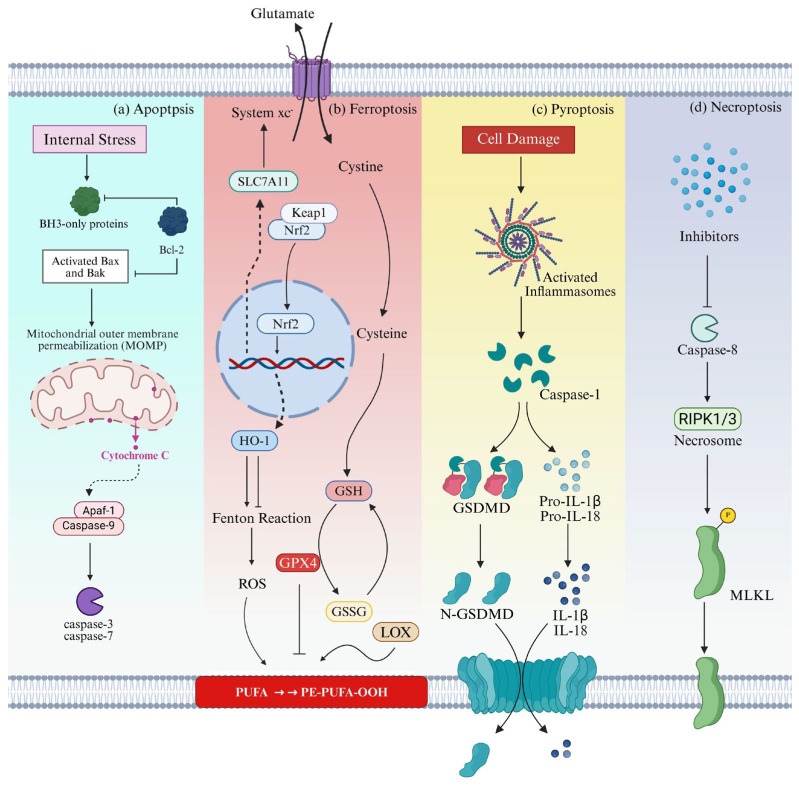
Molecular mechanisms of cell death (**a**) Apoptosis, (**b**) Ferroptosis, (**c**) Pyroptosis, (**d**) Necroptosis. Arrows indicate stimulatory interactions, with solid arrows representing direct effects and dashed arrows representing indirect effects; bars denote inhibitory effects. Cyan background represents apoptosis; red background represents ferroptosis; yellow background represents pyroptosis; purple background represents necroptosis. Created in Biorender. Yuhan Qiao (2026) https://BioRender.com/38cy1ik (accessed on 4 June 2026).

The development of the nervous system is inseparable from cell death [63]. Under normal conditions, programmed cell death eliminates redundant structures; however, under pathological conditions such as oxidative stress and inflammation, the CNS undergoes various forms of cell death, including apoptosis, pyroptosis, ferroptosis, and necroptosis, ultimately leading to neuronal injury and functional impairment. (Figure 10).

In normal CNS cells, the anti-apoptotic protein Bcl-2, a member of the Bcl-2 family, can inhibit the pro-apoptotic effector proteins Bax and Bak, preventing them from initiating cell death. Under pathological conditions, the expression of Bcl-2 family proteins becomes dysregulated, and the loss of Bcl-2-mediated inhibition allows Bax and Bak to increase mitochondrial outer membrane permeability, leading to the release of cytochrome c and activation of the caspase cascade, with caspase-3 ultimately executing cellular disassembly [64,65]. Ferroptosis is driven by iron overload and lipid peroxidation. CNS cells, characterized by high oxygen consumption and abundant polyunsaturated fatty acids, are particularly susceptible to ferroptosis. Under physiological conditions, cells rely on the System Xc^−^-GSH-GPX4 axis to reduce lipid peroxides to non-toxic lipid alcohols and maintain iron homeostasis via iron chelation, thereby suppressing ROS accumulation. However, under pathological states, iron overload or the failure of the antioxidant defense axis allows intracellular free iron to catalyze the generation of massive amounts of ROS via the Fenton reaction, triggering a chain reaction of lipid peroxidation and ultimately leading to ferroptosis [66,67]. Pyroptosis is a pro-inflammatory form of programmed cell death mediated by the NLRP3 inflammasome signaling pathway (see Section 2.3.1), ultimately contributing to neuronal loss and disease progression in CNS diseases [68]. Furthermore, when Caspase-8 is inhibited, necroptosis is initiated by phosphorylation-driven activation of the RIPK1/RIPK3/MLKL signaling axis, resulting in loss of membrane integrity and the release of DAMPs [69]. Therefore, directly targeting the downstream effector molecules of these cell death pathways has emerged as the last line of defense to rescue damaged neurons and restore neural homeostasis.

#### 2.5.2. Novel Cell Death Inhibitors

##### Metformin

Sritawan et al. [70] demonstrated that metformin could significantly antagonize the methotrexate (MTX)-induced decreases in neural stem cell markers Sox2 and nestin in the rat hippocampus, increase levels of the synaptic protein PSD95, and modulate the expression of apoptosis-related proteins, including downregulating caspase-3 and Bax while upregulating Bcl-2. Meanwhile, metformin enhanced the expression of the transcription factor CREB and its phosphorylated form pCREB, thereby promoting neurogenesis, inhibiting apoptosis, and enhancing synaptic plasticity through multiple pathways. In MTX-treated rats, metformin (200 mg/kg) treatment significantly restored PSD95 levels, reduced Caspase-3 content and Bax/Bcl-2 ratio, and restored the expression of proteins such as pCREB/CREB to near-normal levels. Moreover, it restored the number of Sox2-positive cells by approximately 1.94-fold and nestin-positive cells by approximately 1.97-fold in the rat hippocampus. These results strongly indicate that metformin exhibits superior neuroprotective activity in alleviating MTX chemotherapy-related neurotoxicity and memory impairment; however, this study does not include animal behavioral assessments, and the underlying molecular mechanisms, as well as the long-term safety profile, remain to be further elucidated.

##### Ferfluor-1

Ferrostatin-1 (Fer-1) is a classic ferroptosis inhibitor; however, it suffers from poor metabolic stability and insufficient BBB permeability. Based on its structure, Yan et al. [71] introduced a 1,3,4-thiadiazole ring to synthesize the derivative Ferfluor-1. In three cancer cell lines (HT1080, OS-RC-2, and SH-SY5Y), Ferfluor-1 exhibited nanomolar inhibitory activity against RSL3-induced ferroptosis, with EC_50_ values of 57 nM, 75 nM, and 2.3 nM, respectively. As a lipophilic radical-trapping antioxidant, Ferfluor-1 inhibited lipid peroxidation by scavenging phospholipid peroxyl radicals. Its mechanism of action was similar to that of Fer-1, presumably involving inhibition of the 15-LOX-2/PEBP1 complex to reduce lipid peroxidation product formation. Compared with Fer-1, Ferfluor-1 showed significantly improved metabolic stability. The stability of Ferfluor-1 in rat microsomes and plasma increased by approximately 8-fold and 40-fold over Fer-1, respectively, demonstrating favorable pharmacokinetic properties and superior BBB permeability (brain-to-plasma ratio = 0.33). In MCAO and MPTP-induced mouse models, Ferfluor-1 (5 mg/kg) significantly decreased MDA levels and restored dopaminergic neuronal function, thereby attenuating neurological damage. Furthermore, due to its unique excited-state intramolecular proton transfer properties, Ferfluor-1 could serve as a ratiometric photoluminescent probe that specifically responds to phospholipid hydroperoxides and peroxynitrite, enabling real-time visualization of ferroptosis in cells and zebrafish. This study not only provides a multifunctional tool compound with both inhibitory and detection capabilities for the diagnosis and treatment of CNS ferroptosis-related diseases, but also opens a new avenue for developing novel theragnostic probes based on ferroptosis inhibitors. Nevertheless, the pharmacokinetic properties of Ferfluor-1 still require further optimization. Moreover, when used as a probe, its near-infrared imaging performance and potential interference resulting from its inhibitory activity during long-term monitoring also warrant further refinement.

##### Biochanin A

Biochanin A (BCA) is a methoxy isoflavone with estrogen-like activity isolated from legumes such as chickpeas and *Trifolium pratense* L. Its development stems from the potential of traditional Chinese medicine-derived monomeric compounds in multi-targeted neuroprotective strategies. Li et al. [72] found that in the early stage of spinal cord injury (SCI), BCA attenuated inflammasome activation and oxidative stress by inhibiting the TLR4/NF-κB/NLRP3 inflammasome signaling pathway and activating the Nrf2/HO-1 antioxidant pathway, thereby promoting autophagy, reducing apoptosis and pyroptosis, and exerting neuroprotective effects. In an SD rat model of SCI, continuous intraperitoneal injection of BCA (40 mg/kg) for 14 days significantly improved motor function, alleviated spinal cord edema, decreased the levels of inflammatory factors and oxidative stress markers, and upregulated antioxidant enzyme activities. In the early stage of SCI, the therapeutic efficacy of BCA was comparable to that of the positive control methylprednisolone (30 mg/kg). These findings suggest that BCA offers a potential multi-target therapeutic strategy for early intervention in SCI. However, current research is still limited to the early stages in animal models. The direct molecular targets of BCA on signaling pathways, as well as its long-term efficacy and safety, remain unclear, and further mechanistic investigation is warranted.

##### Morin Hydrate

Morin hydrate (MH) is a natural flavonoid whose neuroprotective effects derive from its multi-target regulatory potential against neuroinflammation, apoptosis, and necroptosis, showing particularly strong efficacy in models of neurodegenerative diseases. Research by Elbaz et al. [73] revealed that MH (20 mg/kg) significantly ameliorated motor dysfunction, alleviated weight loss, and reversed striatal histopathological damage in a rat model of Huntington’s disease (HD) induced by 3-nitropropionic acid (3-NP), exhibiting comparable efficacy to the positive control drug necrosulfonamide (NSA). At the molecular level, it downregulated the 3-NP-induced TNF-α expression to 69%, reduced the expression of phosphorylated RIPK1, RIPK3, and MLKL by over 20%, and significantly inhibited caspase-3 activity, while increasing striatal succinate dehydrogenase (SDH) activity by 2.4-fold. Through these actions, it exerted multiple neuroprotective effects, including anti-necroptosis, anti-apoptosis, and anti-neuroinflammation. This study is the first to demonstrate that MH protects the striatum from 3-NP-induced neurotoxicity by modulating the necroptosis pathway involving RIPK1, RIPK3, and MLKL, thereby conferring protection against HD. Future studies should further investigate its pharmacokinetics, safety, and clinical translational potential.

Table 5 summarizes the effects of novel anti-cell death agents, their molecular mechanisms, and their reported activities in various in vivo models for the treatment of CNS diseases.

**Figure 11:**
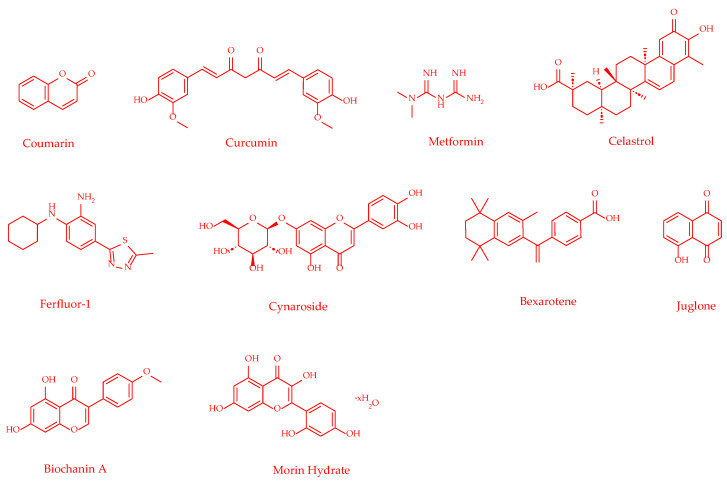
Chemical structures of anti-cell death agents.

Figure 11 shows chemical structures of anti-cell death agents listed in Table 5.

## 3. Challenges and Emerging Strategies

As of 2021, the global number of patients with dementia reached 57 million, with nearly 10 million new cases emerging annually [79]; stroke, as the second leading cause of death and a primary cause of disability worldwide, also exhibits an upward trend in its incidence rate [80]. With the accelerating process of population aging, the incidence of CNS diseases continues to increase, posing severe challenges to the global public health system [81]. Despite the widespread impact of CNS diseases, the costs of corresponding drug research and development (R&D) remain persistently high. On average, it takes over a decade for a new drug to progress from discovery to market, with an estimated total cost of $2.87 billion (in 2013 US dollars) [82]. Due to the unique challenges faced in the treatment of CNS diseases, drug development in this field carries even higher risks and failure rates, and the actual costs far exceed this average.

The first major challenge in CNS drug development is the inadequacy of disease models. Traditionally, rodent models have been predominantly used for CNS drug discovery; however, their CNS differs significantly from that of humans in terms of cellular composition, morphology, and distribution. Furthermore, rodent neurons exhibit apparent resistance to neurodegeneration, thereby limiting their ability to fully recapitulate the pathological features of human neurodegenerative diseases. Although the CNS of non-human primates is more similar to that of humans, the time and financial costs associated with such research are prohibitively high [83]. The emergence of induced pluripotent stem cell (iPSC) technology has marked a turning point in addressing this dilemma. On the one hand, iPSCs can provide neurons and glial cells with specific human genetic backgrounds, enabling researchers to directly verify the therapeutic efficacy and potential toxicity of drugs in a human-derived system, thereby substantially enhancing the efficiency of compound screening. On the other hand, iPSCs themselves possess the therapeutic potential to differentiate into specific cells and replace diseased cells in patients, representing a promising strategy to overcome limitations in neuronal regeneration [84]. Furthermore, iPSC technology can be integrated with microfluidic chip technology to construct organ-on-a-chip models that more closely mimic physiological human conditions, overcoming the limitations of traditional animal models and two-dimensional cell cultures in CNS disease research [85]. Brain organoid technologies developed by researchers can induce iPSCs to self-organize in vitro into three-dimensional neural structures, simulating the cell types, tissue architecture, and partial functions during early human brain development. This technology has already been applied in drug screening and therapeutic exploration for various CNS diseases, such as AD and PD [86,87]. The widespread application of iPSC technology has opened new avenues for constructing more complex drug screening platforms and developing effective cell replacement therapies [84].

The second major challenge in CNS drug development is the low permeability of compounds across the BBB. This natural barrier prevents over 98% of small-molecule drugs from entering the brain, dramatically increasing the difficulty of CNS drug R&D [88]. In recent years, the development of nanodrug delivery systems has demonstrated tremendous potential in addressing this challenge. Nanoparticles within these systems can enrich drug molecules and protect their active functional groups, precisely delivering drugs across the BBB to specific sites for targeted therapy [89]. The use of nanoscale delivery systems can improve drug stability and loading capacity while enhancing targeting efficiency; this not only facilitates the better passage and functioning of compounds across the BBB, but also enables the co-delivery of drugs, imaging agents, and targeting ligands [90]. Regarding BBB permeability assessment, three-dimensional BBB models based on hydrogels, spheroids, and organoids, as well as microfluidic models, can be developed to screen the ability of drug delivery systems to cross the BBB, thereby accelerating the clinical translation of nanodrugs for brain-targeted delivery [91]. Additionally, intranasal administration is gaining increasing attention as an emerging strategy that bypasses the BBB and delivers drugs directly to the brain. Drugs absorbed through the nasal mucosa can directly enter the systemic circulation, effectively circumventing first-pass metabolism in the liver and gastrointestinal tract and significantly improving bioavailability, providing a promising solution to overcome BBB restrictions [92].

The third major challenge in CNS drug development is the high attrition rate and efficiency bottlenecks in translating laboratory lead compounds into clinical applications. Due to the high complexity of the CNS, the low selective permeability of the BBB, and the limitations of traditional pharmacokinetic evaluation models, a large number of candidate molecules with good in vitro activity often fail in the clinical stage because of poor permeability, off-target toxicity, or metabolic instability, leading to prolonged R&D cycle and persistently high costs. With the deeper integration of biology and computer science, artificial intelligence (AI) technology has emerged as a novel strategy to resolve this dilemma, injecting new momentum into the treatment of CNS diseases. At the compound screening stage, machine learning-based AI methods can rapidly screen massive compound libraries to identify molecules capable of modulating key CNS disease targets [93]. In the drug property evaluation phase, machine learning models such as graph neural networks (GNNs) are widely utilized to predict the absorption, distribution, metabolism, excretion, and toxicity of small molecules, thereby eliminating candidates with poor pharmacokinetic properties at an early stage [94]. Concurrently, AI-assisted design of delivery systems is gaining traction: by predicting the optimal size, surface charge, and ligand modifications of nanoparticles, machine learning models can optimize encapsulation efficiency of active molecules in carriers such as liposomes and micelles, achieving precise cross-barrier delivery and controlled release [95]. This full-process AI integration, spanning from molecular design to targeted delivery, is significantly shortening the R&D cycle for CNS drugs, paving the way for innovative neuroprotective therapies, and accelerating drug screening and clinical translation.

## Figures and Tables

**Table 1:** Novel VGIC modulators for CNS diseases.

Lead Compounds	Molecular Mechanism	IC_50_/EC_50_	In Vivo Effect	In Vivo Models	Related Diseases	Ref.
XPC-6591	Bound with high selectivity to the VSD4 domain of Nav1.6, inhibiting Nav1.6 activity	hNaᵥ1.6 IC_50_ = 0.0056 μM; maintains 700-fold and 100-fold selectivity over Nav1.1 and Nav1.5, respectively; brain EC_50_ in Scn8a^+/−^ mice = 0.0047 μM; brain EC_50_ in WT mice (DC-MES) = 0.01 μM	Effectively suppressed seizures in Scn8a^+/−^ mice and electrically stimulated WT mice; due to its high selectivity, no motor coordination impairment or cardiotoxicity was observed.	Scn8a^+/−^ mice; WT mice	Human Scn8a developmental and epileptic encephalopathy; idiopathic epilepsy	[9]
15b	State-dependently bound to the VSD4 domain of inactivated Nav1.3 channels, inhibiting Nav1.3 activity	IC_50_ = 20 nM, maintained 150-fold and 350-fold selectivity over Nav1.5 and Nav1.7, respectively	N/A	N/A	Neuropathic pain	[13]
ICA-27243	Bound to the S1–S4 voltage-sensing domain of the Kv7.2 channel, allosterically activated Kv7.2/Kv7.3 channels, and enhanced M-currents	N/A	Significantly improved neuromuscular function, preserved motor coordination, reduced spinal motor neuron loss, attenuated glial reactivity, and delayed disease onset in mice.	SOD1^G93A^ mice	Amyotrophic lateral sclerosis (ALS)	[11]
Magnolol	Bound to and inhibited multiple T-type calcium channel subtypes (Cav3.1, Cav3.2, Cav3.3) without acting on CB1 or CB2 receptors	Cav3.1 IC50 = 4 μM; Cav3.2 IC50 = 5 μM; Cav3.3 IC50 = 4 μM;	Significantly increased the febrile seizure threshold and protected 90% of Scn1a^+/−^ mice from hindlimb extension; reduced the frequency and duration of atypical absence seizures in Gabrb3^+/D120N^ mice; exhibited excellent brain permeability with a brain-to-plasma ratio of 3.55 in healthy mice.	Scn1a^+/−^ mice; Gabrb3^+/D120N^ mice; WT mice	Epilepsy	[12]
Cinnarizine	Bound to and inhibited T-type Cav3 channels without affecting dopamine (DA) release at low dosage (10 mg/kg)	N/A	Attenuated calcium overload-induced mitochondrial stress and oxidative damage, and protected nigral TH-positive neurons in rats; acted as a DA receptor antagonist (Ki = 13.2 nM), preventing striatal DA depletion.	Lactacystin-induced rats	Parkinson’s disease (PD)	[14]

Abbreviations: N/A, not applicable (due to the lack of specific information in the report).

**Table 2:** Novel excitotoxicity inhibitors for CNS diseases.

Lead Compounds	Molecular Mechanism	IC_50_/EC_50_	In Vivo Effect	In Vivo Models	Related Diseases	Ref.
Compound 2	Bound to the GluN1/2B subunits of NMDAR with high selectivity, antagonizing NMDAR activity; inhibited hAChE activity	NMDAR IC_50_ = 1.32 μM, 11-fold increase in selectivity over the parent compound; hAChE IC_50_ = 0.115 μM, 10-fold increase in inhibitory activity compared to the parent compound; BChE IC_50_ = 0.421 μM	Exhibited a shorter half-life, higher clearance rate, lower volume of distribution, and an over two-fold increase in brain uptake in vivo compared with the parent compound.	WT mice	AD	[24]
THSG	Directly bound to and activated the GluN2B subunit of NMDAR (binding energy = −5.2 kcal/mol)	N/A	Significantly reduced cerebral infarct volume, decreased neurological deficit scores, and inhibited neuronal apoptosis in rats.	tMCAO/R rats	CIRI	[21]
SHPL-49	Upregulated the glutamate transporter GLT-1 in astrocytes, accelerating the clearance of excessive glutamate from the synaptic cleft; specifically bound to and activated the GluN2A subunit of neuronal NMDARs	N/A	Activated the downstream NR2A–CAMKIIα–Akt/CREB pro-survival signaling pathway; decreased glutamate levels in the rat brain, reduced neuronal death, reduced cerebral infarct volume, decreased the modified neurological severity scores (mNSSs), and extended the therapeutic time window.	Permanent middle cerebral artery occlusion (pMCAO) rats	Acute ischemic stroke (AIS)	[25,26]
L-theanine	Bound to and inhibited the overactivation of AMPAR (especially GluA1 subunit); entered astrocytes to promote GSH synthesis.	N/A	Significantly attenuated Ca^2+^/Zn^2+^ accumulation, oxidative stress, and neuroinflammation in the rat hippocampus, reduced neuronal death, and improved cognitive deficits.	CCI-TBI rats	TBI	[23]
Xanthohumol	Competitively bound to AMPAR (GluA1/GluA3 subunits) and NMDAR (GluN1/GluN2A subunits) with glutamate; regulated peripheral metabolism, thereby reducing glutamate synthesis in the blood and intestines	N/A	Upregulated the expression of key proteins in mitochondrial oxidative phosphorylation complexes in mice, reduced excitotoxicity in glutamatergic neurons, downregulated apoptotic signaling in hippocampal neurons, and moderately improved spatial learning and memory.	APP/PS1 mice	AD	[27]

Abbreviations: N/A, not applicable (due to the lack of specific information in the report).

**Table 3:** Novel anti-inflammatory agents for CNS diseases.

Lead Compounds	Molecular Mechanism	IC_50_/EC_50_	In Vivo Effect	In Vivo Models	Related Diseases	Ref.
NT-0796	NDT-19795 (the active metabolite of NT-0796) directly bound to the NACHT domain of the NLRP3 protein, thereby inhibiting NLRP3 inflammasome activation	PBMC IL-1β IC_50_ = 0.32 nM; whole blood IL-1β IC_50_ = 6.8 nM; ASC speck IC_50_ = 2.1 nM	Inhibited NLRP3-dependent IL-1β release in human PBMCs and whole blood; demonstrated a brain-to-plasma distribution ratio of 0.79 in WT mice, indicating effective BBB penetrability; reduced systemic inflammatory markers in PD patients with favorable safety, tolerability, and minimal adverse events.	WT mice; Clinical samples from PD patients	PD	[35,36]
Rebamipide	Directly bound to the NLRP3-NEK7 complex, thereby blocking NLRP3 inflammasome assembly	N/A	Inhibited microglial activation and pro-inflammatory cytokine release, attenuated dopaminergic neuron loss, and improved motor function in mice.	MPTP-induced mice	PD	[37]
Talniflumate	Blocked the physical interaction between ASCT2 and NLRP3 in astrocytes, thus inhibiting NLRP3 inflammasome assembly and activation	N/A	Effectively penetrated the murine BBB, alleviated neuroinflammation, protected dopaminergic neurons, and significantly improved motor function.	MPTP-induced mice	PD	[32]
Metformin	Activated the Nrf2-ARE antioxidant signaling pathway and inhibited the NF-κB inflammatory pathway	N/A	Significantly attenuated neuroinflammatory responses in mice following stroke and TS exposure, reducing levels of multiple inflammatory cytokines.	Adolescent mice (14-day tobacco smoke inhalation)	IS	[33]
3-Monothiopomalidomide	Bound to CRBN and interfered with inflammatory signal transduction	IC_50_ = 0.21μM	Exhibits a brain-to-plasma concentration ratio of 0.44–0.47 in WT mice; reduced pro-inflammatory cytokine and chemokine levels without decreasing levels of IL-10; significantly alleviated behavioral deficits and reduced astrocyte and microglial activation in CCI-TBI mice; did not trigger teratogenic adverse effects.	WT mice; LPS-induced mice; CCI-TBI mice; Chicken embryos	TBI	[34]
H_2_S	Downregulated the expression of the chemokine CXCL12	N/A	Reduced macrophage infiltration density in tumor tissues, decreased CXCL12 mRNA and protein expression levels in tumor cells and tissues, and significantly prolonged survival of patients and mice.	Immunocompetent Nestin-TVA genetically engineered mice; Surgical samples from IDH wildtype glioblastoma patients	IDH wildtype glioblastoma	[38]
A14	Bound to CCR5, thereby blocking its interaction with the chemokine-like factor CKLF1	IC_50_ = 4.29 μM	Significantly attenuated neuroinflammation in mice, inhibited glial cell activation, reduced peripheral immune cell infiltration; compared with the positive control drug maraviroc, A14 more effectively improved motor function and exhibited superior BBB permeability.	Photothrombotic Stroke (PTS) mice	IS	[39]

Abbreviations: N/A, not applicable (due to the lack of specific information in the report).

**Table 4:** Novel antioxidants for CNS diseases.

Lead Compounds	Molecular Mechanism	IC_50_/EC_50_	In Vivo Effect	In Vivo Models	Related Diseases	Ref.
Puerarin	Inhibited the activation of DNA methyltransferases 3A and 3B, induced demethylation of the PI3K promoter, and activated the PI3K/Akt pathway	N/A	Lowered neurological deficit scores, attenuated neuronal injury, reduced the neuronal apoptosis rate in ischemic brain tissue from 48.5% to 19.7%, decreased cerebral infarct volume, and alleviated oxidative stress-induced injury in MCAO/R rats; increased the survival rate of transient middle cerebral artery occlusion (tMCAO) rats from 60% to 75%.	MCAO/R rats; tMCAO rats	CIRI	[50,51]
Kaempferol	Bound to and inhibited Cal3 expression (- CDOCKER interaction energy = 57.2532); activated the PI3K/Akt pathway	N/A	Upregulated downstream antioxidant pathways, significantly reduced cerebral infarct volume in mice, inhibited neuroinflammation and oxidative stress, and improved sensorimotor functions.	PTS mice	IS	[52]
Gomisin N	Promoted the phosphorylation of PI3K, Akt, and mTOR proteins (binding energy: PI3K = −9.9 kcal/mol; Akt = −8.5 kcal/mol; mTOR = −8.0 kcal/mol), thereby activating the PI3K/Akt/mTOR pathway	N/A	Significantly attenuated neuronal injury in mice, reduced cerebral infarct volume, and improved neurological deficits.	MCAO/R mice	IS	[44]
Lyoniresinol	Activated the PI3K/Akt/GSK-3β/Nrf2 signaling pathway	N/A	Significantly decreased neurological function scores, reduced cerebral infarct volume, attenuated pathological damage in hippocampal CA1/CA3/DG regions, decreased MDA content, and elevated antioxidant enzyme levels in rats.	middle cerebral artery occlusion (MCAO) rats	IS	[53]
Loureirin C	Promoted the dissociation of Nrf2 from Keap1 and its translocation to the nucleus	N/A	Dose-dependently alleviated neurological deficits, reduced cerebral infarct volume, decreased iron accumulation, lipid peroxidation, and ROS levels, and inhibited ferroptosis in mice; these effects were attenuated upon Nrf2 knockdown.	MCAO/R mice	IS	[54]
Atractylenolide III	Directly targeted and stably bound to the Kelch domain of the Keap1 protein (binding energy = −9.4 kcal/mol), disrupting the Keap1-Nrf2 complex	N/A	Dose-dependently reduced cerebral infarct volume from 42% to 20%, improved neurological deficits, and exhibited favorable BBB permeability and safety in mice.	MCAO/R mice	IS	[55]
Caffeic acid	Activated the Nrf2 signaling pathway	N/A	Significantly reduced cerebral infarct volume and oxidative stress marker levels, increased antioxidant enzyme content, and improved neurological deficits in rats; exhibited a therapeutic window of up to 2 h post-ischemia.	pMCAO rats	IS	[56]
NBP	Activated the Nrf2/ARE signaling pathway	N/A	Significantly reduced oxidative stress and inflammation levels, ameliorated photophobic and anxiety-like behaviors in NTG-induced mice; compared with edaravone-dexborneol, NBP exhibited superior efficacy in reducing cerebral infarct volume, restoring cerebral blood flow, and scavenging oxidative stress factors in MCAO/R rats.	NTG-induced mice; MCAO/R rats	Chronic migraine	[46,47]
8a	Bound to Keap1 (K_D_ = 561 nM), promoting Nrf2 dissociation and nuclear translocation	N/A	8a (20 mg/kg) significantly reduced cerebral infarct volume, decreased neuronal apoptosis and ferroptosis, and improved neurological function scores in rats; therapeutic efficacy was superior to an equivalent dose of NBP.	MCAO/R rats	IS	[48]
Chlorogenic Acid	Directly scavenged or inhibited excessive ROS production; maintained Akt phosphorylation levels and suppressed Erk1/2 hyperactivation	N/A	Blocked neuronal apoptosis mediated by the Akt/Erk1/2 pathway, improved motor coordination and gait parameters, prevented the decrease in tyrosine hydroxylase expression in the substantia nigra-striatum, and reduced p-Erk1/2 levels.	A53T mice	PD	[57]
Cycloastragenol	Inhibited the interaction between the PDZ domain of Scrib and the NOX subunit p22^phox^ in microglia, suppressing NOX activation; enhanced autophagy, promoted the clearance of α-Syn and damaged mitochondria, and inhibited NLRP3 activation	N/A	Significantly reduced α-syn aggregation in the nigrostriatal region, preserved dopaminergic neurons, improved motor coordination and anxiety-like behaviors in mice.	AAV2/9-hSyn-SNCA(A53T)-α-Syn-WPRE injected mice	PD	[58]
Avicularin	Directly bound to NOX4 (molecular docking binding energy −8.52 kcal/mol, K_D_ = 37.97 μM)	N/A	Improved cognitive function, restored antioxidant enzyme levels, inhibited ferroptosis, protected neurons, suppressed astrocyte hyperactivation in both AD models, and reduced Aβ plaque deposition in APP/PS1 mice.	SCOP-induced mice; APP/PS1 mice	AD	[59]
Crocetin	Inhibited the interaction between PKC ζ and the NOX subunit p47^phox^, blocking the assembly and activation of the NOX2 complex in the early phase; directly bound to HK-I, inhibiting apoptosis in the late phase.	N/A	Reduced cerebral infarct volume from 43.11% to 12.91% and mNSS from 13.2 to 9.4, decreased oxidative stress markers such as MDA and ROS, and inhibited HK-I-mediated parthanatos in rats.	pMCAO rats	IS	[49]
Crebanine	Downregulated the expression of NOX2 subunits gp91^phox^ and p47^phox^, inhibiting NOX2 activation	N/A	Significantly ameliorated cerebral edema, infarct volume, and neurological deficits in rats; inhibited NF-κB and MAPK pathways, and reduced the expression of inflammatory cytokines such as TNF-α and IL-1β.	MCAO/R rats	Ischemia–reperfusion (I/R)	[60]
NCATS-SM7270	Inhibited NOX2 expression	Human neutrophil NOX2 IC_50_ = 4.09 μM; mice bone marrow granulocyte NOX2 IC_50_ = 4.28 μM	Dose-dependently reduced cortical cell death in mice, with efficacy comparable to NOX2 gene knockout.	mild traumatic brain injury (mTBI) mice	TBI	[61]
Compound **17**	Competitively occupied the L-arginine binding site of nNOS, inhibiting nNOS catalytic activity	Rat nNOS K_i_ = 15 nM; human nNOS K_i_ = 19 nM, maintains 1075-fold and 115-fold selectivity over eNOS and iNOS, respectively	N/A	N/A	Neurodegenerative Diseases	[62]

Abbreviations: N/A, not applicable (due to the lack of specific information in the report).

**Table 5:** Novel anti-cell death agents for CNS diseases.

Lead Compounds	Molecular Mechanism	IC_50_/EC_50_	In Vivo Effect	In Vivo Models	Related Diseases	Ref.
Curcumin + Ginkgo biloba extract (GBE)	GBE promoted the brain distribution of curcumin by regulating BBB permeability, reducing caspase-3-mediated apoptosis	N/A	Significantly ameliorated cognitive dysfunction, hippocampal neurodegeneration, and the accumulation of intracellular neurofibrillary tangles (NFTs) and extracellular Aβ plaques in rats; the combined treatment demonstrated superior efficacy compared with curcumin alone.	SCO/HMM-induced rats	AD	[74]
Metformin	Increased hippocampal neural stem cell markers; reduced apoptosis markers; restored the synaptic protein PSD95; activated CREB/pCREB transcriptional signaling.	N/A	Significantly restored PSD95 levels, reduced apoptosis marker levels, and restored proteins such as pCREB/CREB to near-normal levels in rats; the number of Sox2-positive and nestin-positive hippocampal cells recovered by approximately 1.94-fold and 1.97-fold in rats, respectively.	MTX-treated rats	MTX chemotherapy-induced cognitive impairment; hippocampal neurogenesis damage	[70]
Celastrol	Upregulated the Nrf2-xCT-GPX4 axis, promoting GPX4 expression	N/A	Promoted recovery of hindlimb motor function in rats, attenuated ferroptosis in spinal cord.	T10 spinal cord contusion rats	SCI	[75]
Ferfluor-1	Inhibited the 15-LOX-2/PEBP1 complex, reducing the production of lipid peroxidation products; scavenged phospholipid peroxyl radicals, thereby inhibiting lipid peroxidation	HT1080 EC_50_ = 57 nM; OS-RC-2 EC_50_ = 75 nM; SH-SY5Y EC_50_ = 2.3 nM;	Exhibited favorable pharmacokinetic properties and BBB permeability (brain-to-plasma ratio = 0.33); significantly attenuated neuronal injury in MCAO and MPTP mice by reducing MDA levels and restoring dopaminergic neuronal function; functioned as a ratiometric photoluminescent probe for real-time visualization of ferroptosis progression in zebrafish.	WT mice; MCAO mice; MPTP-induced mice; Zebrafish	IS; PD	[71]
Cynaroside	Activated the IRF1/SLC7A11/GPX4 signaling pathway, promoting GPX4 expression; inhibited microglial M1 polarization and inflammatory responses	N/A	Effectively ameliorated depressive-like behaviors in mice, attenuated hippocampal inflammation and neuronal injury, with efficacy comparable to the positive control fluoxetine.	Chronic unpredictable mild stress (CUMS) mice	Depression	[76]
Bexarotene	Activated the AMPK-mTOR-TFE3 axis, thereby promoting autophagy, reducing ROS levels, inhibiting NLRP3 activation, and downregulating the NLRP3-GSDMD pathway	N/A	Reduced spinal oxidative stress markers, decreased the expression of pyroptosis-related proteins, promoted autophagy, and facilitated motor function recovery in mice.	T11–T12 spinal cord contusion mice	SCI	[77]
Juglone	Downregulated the expression of the transcription factor FOS, thereby reducing USP53 transcription and enhancing the ubiquitin-dependent degradation of the pyroptosis executioner protein GSDMD and the necroptosis executioner protein MLKL	N/A	Alleviated neuronal necroptosis and promoted functional recovery after SCI in mice.	T10 spinal cord contusion mice	SCI	[78]
Biochanin A	Inhibited the TLR4/NF-κB/NLRP3 signaling pathway; activated the Nrf2/HO-1 signaling pathway	N/A	Significantly improved motor function, attenuated spinal cord edema, reduced levels of inflammatory cytokines and oxidative stress markers, upregulated antioxidant enzyme activity, decreased neuronal apoptosis and pyroptosis in rats; in early-stage SCI treatment, its efficacy was comparable to methylprednisolone.	Weight-drop method-induced SCI rat model	SCI	[72]
Morin hydrate	Downregulated the expression of phosphorylated RIPK1, RIPK3, and MLKL by over 20%; suppressed the release of inflammatory cytokines, reducing TNF-α expression to 69%; significantly inhibited caspase-3 activity	N/A	Significantly ameliorated motor dysfunction in rats, alleviated weight loss, and reversed striatal histopathological damage; decreased apoptosis and necroptosis markers, and increased SDH activity.	3-NP-induced HD rats	HD	[73]

Abbreviations: N/A, not applicable (due to the lack of specific information in the report).

## Data Availability

No new data were created or analyzed in this study. Data sharing is not applicable.

## Abbreviations

The following abbreviations are used in this manuscript:

CNS	Central nervous system
IS	Ischemic stroke
TBI	Traumatic brain injury
AD	Alzheimer’s disease
VGIC	Voltage-gated ion channel
VGSC	Voltage-gated sodium channel
VGKC	Voltage-gated potassium channel
VGCC	Voltage-gated calcium channel
VSD4	Voltage-sensing domain 4
DE-MES	Direct current multielectrode shock
WT	Wild-type
MES	Maximal electroshock
ALS	Amyotrophic lateral sclerosis
PD	Parkinson’s disease
DA	Dopamine
EAAT	Excitatory amino acid transporter
MAPK	Mitogen-activated protein kinase
NMDAR	N-methyl-D-aspartate receptor
AMPAR	α-amino-3-hydroxy-5-methyl-4-isoxazolepropionic acid receptor
THSG	Tetrahydroxystilbene-2-O-β-D-glucoside
CI/RI	Cerebral ischemia–reperfusion injury
tMCAO/R	Transient middle cerebral artery occlusion/reperfusion
CCI-TBI	Controlled cortical impact—traumatic brain injury
mNSS	Modified neurological severity scores
pMCAO	Permanent middle cerebral artery occlusion
AIS	Acute ischemic stroke
PAMPs	Pathogen-associated molecular patterns
DAMPs	Damage-associated molecular patterns
GSDMD	Gasdermin D
BBB	Blood–brain barrier
ASCT2	Alanine-serine-cysteine transporter 2
MF	Metformin
TS	Tobacco smoke
3-MP	3-Monothiopomalidomide
CRBN	Cereblon
PTS	Photothrombotic stroke
ROS	Reactive oxygen species
RNS	Reactive nitrogen species
NOX	NADPH oxidase
NO	Nitric oxide
NOS	Nitric oxide synthases
HO-1	Heme oxygenase-1
GN	Gomisin N
MCAO/R	Middle cerebral artery occlusion/reperfusion
NBP	Dl-3-n-butylphthalide
NTG	Nitroglycerin
TMP	Ligustrazine
PKCζ	Protein kinase Cζ
HK-1	Hexokinase-I
AIF	Apoptosis-inducing factor
tMCAO	Transient middle cerebral artery occlusion
MCAO	Middle cerebral artery occlusion
IR	Ischemia–reperfusion
mTBI	Mild traumatic brain injury
MTX	Methotrexate
Fer-1	Ferrostatin-1
BCA	Biochanin A
SCI	Spinal cord injury
MH	Morin hydrate
3-MP	3-nitropropionic acid
HD	Huntington’s disease
NSA	Necrosulfonamide
SDH	Succinate dehydrogenase
NFTs	Neurofibrillary tangles
GBE	Ginkgo biloba extract
CUMS	Chronic unpredictable mild stress
iPSC	Induced pluripotent stem cell
R&D	Research and development
AI	Artificial intelligence
GNN	Graph neural network
